# Effect of Preoperative Urodynamic Study on Urinary Outcomes after Transobturator Sling

**DOI:** 10.1055/s-0040-1719148

**Published:** 2021-01-19

**Authors:** Pedro Rincon Cintra da Cruz, Aderivaldo Cabral Dias Filho, Gabriel Nardi Furtado, Rhaniellen Silva Ferreira, Ceres Nunes Resende

**Affiliations:** 1 Hospital Universitário de Brasília, Brasília, DF, Brazil; 2 Hospital de Base do Distrito Federal, Brasília, DF, Brazil; 3 Universidade Estadual de Campinas, Campinas, SP, Brazil

**Keywords:** urinary incontinence, urodynamic, stress urinary incontinence, transobturator suburethral tape, propensity score, incontinência urinária, urodinâmica, incontinência urinária de esforço, sling suburetral transobturatório, pontuação de propensão

## Abstract

**Objective** To evaluate whether performing preoperative urodynamic study influences postoperative urinary symptoms of women with stress urinary incontinence that underwent transobturator sling.

**Methods** Retrospective analysis of patients treated for stress urinary incontinence by transobturator sling from August 2011 to October 2018. Predictor variables included preoperative urodynamic study, age, incontinence severity, body mass index, preoperative storage symptoms and previous anti-urinary incontinence procedure. Outcome variables were postoperative subjective continence status, storage symptoms and complications. Logistic regression after propensity score was employed to compare outcomes between patients who underwent or not pre-operative urodynamic study.

**Results** The present study included 88 patients with an average follow-up of 269 days. Most patients ( *n =* 52; 59.1%) described storage symptoms other than stress urinary incontinence, and 38 patients (43.2%) underwent preoperative urodynamic studies. Logistic regression after propensity score did not reveal an association between urinary continence outcomes and performance of preoperative urodynamic study (odds ratio 0.57; confidence interval [CI]: 0.11–2.49). Among women that did not undergo urodynamic study, there was a subjective improvement in urinary incontinence in 92% of the cases versus 87% in those that underwent urodynamic study ( *p* = 0.461). Furthermore, postoperative storage symptoms were similar between women who did not undergo urodynamic study and those who underwent urodynamic study, 13.2% versus 18.4%, respectively ( *p* = 0.753).

**Conclusion** Preoperative urodynamic study had no impact on urinary incontinence cure outcomes as well as on urinary storage symptoms after the transobturator sling in women with stress urinary incontinence.

## Resumo

**Objetivo** Avaliar a influência do estudo urodinâmico pré-operatório nos resultados miccionais pós-operatórios em mulheres com incontinência urinária de esforço submetidas a sling transobturador.

**Métodos** Análise retrospectiva de mulheres com incontinência urinária de esforço submetidas a sling transobturador entre agosto de 2011 e outubro de 2018. As variáveis preditoras pré-operatórias, entre outras, foram a realização do estudo urodinâmico, gravidade da incontinência e sintomas urinários de armazenamento. As variáveis de desfecho pós-operatórias foram o status subjetivo da continência, sintomas de armazenamento urinário e complicações cirúrgicas. A regressão logística após o escore de propensão foi empregada para comparar os resultados entre os pacientes que foram submetidos ou não ao estudo urodinâmico pré-operatório.

**Resultados** Foram incluídas no presente estudo 88 pacientes com um seguimento médio de 269 dias. A maioria das pacientes apresentava sintomas miccionais de armazenamento ( *n =* 52; 59,1%) concomitantes à incontinência urinária de esforço. Um pouco menos da metade das pacientes ( *n =* 38; 43,2%) foram submetidas a estudo urodinâmico pré-operatório. A regressão logística após o escore de propensão não revelou associação entre os resultados de continência urinária e a realização de estudo urodinâmico pré-operatório (odds ratio 0,57; intervalo de confiança [IC]: 0,11–2,49). Além disso, os sintomas de armazenamento urinário pós-operatórios foram similares entre as pacientes que não realizaram e aquelas que realizaram o estudo urodinâmico, 13,2% e 18,4% respectivamente ( *p* = 0,753).

**Conclusão** O estudo urodinâmico pré-operatório não teve impacto nos resultados de continência urinária, bem como nos sintomas de armazenamento urinário após o sling transobturatório.

## Introduction

Urinary incontinence (UI) is an important cause of social isolation and poor quality of life among women. This is a common condition, with prevalence ranging from 15.7 to 49.6%. 1 2 About half of incontinent women have stress urinary incontinence (SUI), which is, therefore, the main cause of UI in this population. 2

Synthetic midurethral slings (MUS) have gradually replaced autologous fascial slings in the treatment of women with SUI. 3 From studies developed by Ulmsten et al. 4 and Petros 5 in the 1990's, the tension-free vaginal tape (TVT) was consolidated as the surgical procedure of choice in women with SUI. In 2001, the transobturator tension-free vaginal tape (TOT) procedure emerged as an effective and technically simpler procedure than TVT with a lower rate of vascular, bladder and intestinal injuries. 6

Urodynamic studies (UDS) are widely performed, but their role in the preoperative assessment of women candidates for the TOT procedure remains in dispute. Guidelines from major urological societies indicate that UDS is not mandatory in women with uncomplicated SUI. 7 8 Some authors, however, justify preoperative UDS in patients with SUI with the argument that it avoids unnecessary operations and provides accurate information to patients about therapeutic outcomes. 9 10 However, there is weak evidence that UDS improve clinical outcomes or that they predict the success of the surgery. 11

Some doubts remain regarding the need to perform the preoperative UDS and their real impact on postoperative urinary outcomes. Our hypothesis is that clinical history and physical examination are sufficient for indicating surgical treatment in most women with UI and stress symptoms predominately. The aim of our study was to evaluate whether performing preoperative UDS influences postoperative urinary symptoms of women with SUI who underwent the TOT procedure.

## Methods

### Study Design and Patient Eligibility

After Institutional Board Review (CAAE: 27824819.3.0000.5558), we retrospectively identified and collected data from all consecutive patients treated for SUI by TOT procedure from August 2011 to October 2018 by the urology and gynecology team at our hospital.

Patient assessment included urogynecological examination and supine stress test with variable bladder volume stress tests. Patients with a high volume of urine loss or in those in whom treatment with perineal physiotherapy was unsuccessful, who wished to progress to surgical treatment, preoperative UDS were performed. However, UDS were not indicated for patients evaluated and operated by a single surgeon who waived them in patients with SUI without prior incontinence surgery. That is, except when evaluated by this surgeon, all other patients underwent UDS before TOT placement.

The TOTs were inserted by the out-inside route under spinal anesthesia. Patients received first generation cephalosporin as a prophylactic antibiotic. A low-cost 20 × 1.5 cm polypropylene mesh, prepared by the surgeon himself, was used in all cases. 12 13 Each end of the mesh was anchored with a zero polypropylene suture used to attach the tape to the helical needle.

Follow-up visits took place 1 month after the procedure and with 3-month intervals during 1 year, and yearly thereafter. In these visits, clinical history was collected and the urogynecological examination and supine stress test was performed.

All patients who underwent TOT from August 2011 to October 2018 were candidates for inclusion in the study, including those with preoperative storage symptoms as well as those who underwent concomitant vaginal prolapse repair. We excluded from analysis patients who did not return for postoperative follow-up in the 1 ^st^ month and those in whom the continence status could not be recovered from their clinical records. Continence status was assessed according to the symptoms of the patient, classified as either cured/improved or unaltered/worsened, and by the average daily number of pads used by the patients

### Study Variables

The predictor variables were: whether the patient underwent or not a preoperative urodynamic study; age in years; incontinence severity according to the average daily number of pads used by the patients (1: ≤ 1 pad/day, 2: 2 or 3 pads/day, 3: > 3 pads/day); vaginal parity, defined as the number of vaginal births; menopause status (yes or no); body mass index (BMI) in Kg/m ^2^ ; preoperative storage symptoms, classified as absent, mild or moderate and whether the patient had a previous anti-urinary incontinence procedure (yes, no).

Outcome variables were postoperative subjective continence status at the last follow-up visit, classified as either cured/improved or unaltered/worsened; postoperative storage symptoms (present or absent) and postoperative complications.

## Statistical Analysis

### Univariate and Bivariate Analysis

Categorical and ordinal variables were described by their frequencies and continuous variables by their medians and interquartile ratios (IQR). Differences between categorical variables were assessed with the Pearson chi-squared and Fisher exact tests, as appropriate. Differences between continuous variables were evaluated with the Kruskal-Wallis test. Where appropriate, statistical significance was set at *p* < 0.05.

### Propensity Score Matching and Logistic Regression

Nearest neighbor propensity score 1:1 matching was employed to compare outcomes between patients that underwent or not preoperative UDS. The variables used to produce propensity scores were age, vaginal parity, preoperative storage symptoms, BMI, and incontinence severity, with missing values imputed via predictive mean matching. Outcomes were assessed by logistic regression models.

### Statistical Software

Statistical analysis was performed within the R language statistical environment (R Foundation, Vienna, Austria). 14

## Results

During the study period, 99 patients underwent the TOT procedure. Eleven patients (11.1%) were excluded due to lack of records for postoperative incontinence status, leaving 88 patients in our study population. The mean postoperative follow-up was of ∼ 269 days.

Overall characteristics of the patients are displayed in Table 1 . The average age of the patients was 52.7 years old and the average BMI was 29.67 Kg/m ^2^ . Most patients ( *n =* 52; 59.1%) described storage symptoms other than SUI. The storage symptoms described were urinary urgency and increased daytime frequency. There were no cases of mixed UI. A greater proportion ( *n =* 75; 85.2%) presented mild stress incontinence, using at most one pad per day. Thirty-eight patients (43.2%) underwent preoperative UDS.

**Table 1:** Overall patient characteristics ( *n* = 88)

Variables		Median (IQR)
Continuous	Age (years old)	51.1 (45.1, 59.4)
	BMI (kg/m ^2^ )	28.2 (26.5, 34.3)
Ordinal	**Vaginal deliveries**	**N (%)**
	0	7(8)
	1–3	51 (58%)
	> 4	30 (34.1%)
	Incontinence severity	
	1	75 (85.2%)
	2	11 (11.4%)
	3	3 (3.4%)
		**N (%)**
**Categorical**	Storage symptoms	52 (59.1%)
	Previous UI surgery	15 (17%)
	DM	10 (11.4%)
	Smoking	14 (15.9%)
	Preoperative UDS	38 (43.2%)
	Synchronous prolapse surgery	32 (36.4%)
	Postoperative continence	80 (90.9%)

Abbreviations: BMI, body mass index; DM, diabetes mellitus; IQR, interquartile ratio; n, number of patients; Preoperative UDS, preoperative urodynamic study; Previous UI surgery, previous urinary incontinence surgery.

The patients who underwent preoperative UDS were not significantly different with respect to age, BMI, number of vaginal deliveries, incontinence severity, and preoperative storage symptoms. However, patients who had previous anti-incontinence surgery were more often submitted to preoperative UDS than those who had no such history (28.9% versus 8.0%, *p* = 0.021). The synchronous prolapse surgeries performed were anterior vaginal repair, posterior vaginal repair and, in some cases, concomitant anterior and posterior repair. The synchronous prolapse surgery performed was similar in the group that underwent UDS and in the group that did not undergo UDS (34.2% versus 38.0%, *p* = 0,087). The distributions of the variables according to the performance or not of preoperative UDS before and after matching are shown in Table 2 .

**Table 2:** Distribution of variables before and after nearest neighbor propensity score matching

	Before matching			After matching		
	No UDS ( *n* = 50)	UDS ( *n* = 38)	p (test)	No UDS ( *n* = 38)	UDS ( *n* = 38)	p (test)
Age (years old)	49.6 (45.1, 56.1)	53.5 (47.4, 61.1)	0.188 (K)	50.60 (46.4, 58.1)	53.5 (47.4, 61.1)	0.496 (K)
BMI (kg/m ^2^ )	29.3 (25.7, 32.3)	28.3 (26.9, 34.7)	0.870 (K)	29.65 (25.6, 34.0)	28.30 (26.9, 34.6)	0.852 (K)
Vaginal deliveries						
0	4 (8%)	3 (7.9%)	0.639 (K)	4 (10.5%)	3 (7.9%)	0.919 (K)
1–3	31 (62%)	20 (52.6%)		19 (50%)	20 (52.6%)	
> 4	15 (30%)	15 (39.5%)		15 (39.5%)	15 (39.5%)	
Incontinence severity						
1	45 (90%)	30 (78.9%)	0.108 (K)	33 (86.8%)	30 (78.9%)	0.208 (K)
2	5 (10%)	5 (13.2%)		5 (13.2%)	5 (13.2%)	
3	0	3 (7.9%)		0	3 (7.9%)	
Menopause	24 (48.8%)	24 (63.2%)	0.231 (C)	19 (50%)	24 (63.2%)	0.355 (C)
DM	3 (6%)	7 (18.4%)	0.139 (F)	3 (7.9%)	7 (18.4%)	0.309 (F)
Smoking	7 (14%)	7 (18.4%)	0.789 (C)	5 (13.2%	7 (18.4%)	0.753 (C)
Previous UI surgery	4 (8%)	11 (28.9%)	0.021 (F)	4 (10.5%)	11 (28.5%)	0.100 (F)
Preoperative storage symptoms	31 (62%)	21 (55.2%)	0.676 (C)	21 (55.3%)	21 (55.3%)	1.0 (C)
Synchronous prolapse surgery	19 (38.0%)	13 (34.2%)	0.887 (C)	13 (34.2%)	13 (34.2%)	1.0 (C)
Postoperative storage symptoms	6 (12.0%)	7 (18.4%)	0.591 (F)	5 (13.2%)	7 (18.4%)	0.753 (F)
Postoperative continence	47 (94.0%)	33 (87%)	0.252 (C)	35 (92.1%)	33 (86.8%)	0.709 (C)

Abbreviations: BMI, body mass index; C, Pearson's Chi-Squared test; DM, diabetes mellitus; F, Fisher exact test; IQR, interquartile ratio; K, Kruskal-Wallis test (followed by Dunn test as appropriate); n, number of patients; Preoperative UDS, preoperative urodynamic study; Previous UI surgery, previous urinary incontinence surgery.

Logistic regression after propensity score did not reveal an association between urinary continence outcomes and performance of preoperative UDS (odds ratio [OR] 0.57; confidence interval [CI]: 0.11–2.49). In addition, among women who did not undergo UDS, there was a subjective improvement in UI in 92% of the cases versus 87% in those who underwent UDS ( *p* = 0.461). The vast majority of patients had no postoperative urinary leakage, 84% (41/50) in the non-UDS group and 76% (29/38) in the UDS group. Postoperative storage symptoms, urinary urgency and increased daytime frequency were similar between women who did not undergo UDS and those who underwent UDS, 13.2% versus 18.4%, respectively ( *p* = 0.753). In addition, there were no cases of de novo storage urinary symptoms among women who did not undergo UDS, and only 2 cases among women who underwent UDS, with no statistical difference between groups ( *p* = 0.474).

In the follow-up, no patient required urethrolysis secondary to urinary retention. There was only one mesh erosion in a patient who underwent a previous urodynamic study and who did not perform prolapse surgery concomitant with the sling.

## Discussion

Our study, which included 88 women with a mean age of 52.7 years old and mean postoperative follow-up of ∼ 269 days, did not indicate an association between the performance of preoperative UDS and urinary continence outcomes for women with SUI. In addition, postoperative storage symptoms were similar between women who did not undergo UDS and those who underwent UDS, 13.2% versus 18.4%, respectively ( *p* = 0.753). It is important to note that 62% of the patients in the group that did not undergo UDS and 55.2% in the group that underwent UDS had preoperative storage symptoms.

Our results are in line with recently published studies. In a large multicenter and randomized study, the Value study, involving women with uncomplicated SUI, whose primary outcome was treatment success, the results of the surgery were compared between those who underwent and those who did not undergo UDS in the preoperative period. The conclusion was that the basic office evaluation is not inferior to that associated with the performance of UDS in the preoperative period. 15 A secondary analysis from the Value study indicated that preoperative UDS in women with uncomplicated SUI increased costs and did not lead to better postoperative outcomes. 16 Rachaneni et al., in a 2015 17 meta-analysis, found that in women with uncomplicated SUI or mixed urinary incontinence with predominantly stress symptoms, with preserved maximal bladder capacity and normal post-void residue, preoperative UDS had no additional value. Finally, recently, a Mayo Clinic retrospective study including 1,629 women submitted to primary synthetic midurethral sling placement did not uncover UDS parameters associated with the necessity for sling release. 18

Despite the results of the studies cited above, many authors have questioned the applicability of these findings in clinical practice. A large Italian multicenter retrospective study of 2,053 women showed that only 36% of the patients with SUI could be classified as uncomplicated SUI. Moreover, even in uncomplicated patients, UDS were able to diagnose voiding dysfunction in 13.4% of the cases and change the management in 11% of the cases. 19 In another Italian study, with a cohort of 323 patients with SUI, the prevalence of uncomplicated SUI was 20.7%. Of these, 11.7% were excluded from the diagnosis of uncomplicated SUI after UDS and 8.96% had the surgical procedure canceled. 20

But is changing the therapeutic strategy of an uncomplicated SUI patient according to urodynamic findings a good strategy? In an interesting multicenter German study, van Leijsen et al. 21 evaluated patients with clinical diagnosis of uncomplicated SUI that differed from urodynamic diagnosis. These women were randomized to surgical or clinical treatment immediately after UDS. The conclusion was that from the point of view of curing UI, the surgical procedure was not inferior to the individualized clinical treatment based on UDS. 21 Furthermore, the effectiveness of TOT procedures has been demonstrated in the literature for a long time. A prospective trial published in 2004 showed an objective cure rate for TOT procedures of 90% and a subjective cure of 86.7%. 22 A systematic review of The Cochrane Database showed subjective cure rates with short, medium and long-term TOT procedures of 83.3%, 86.9% and 84.3%, respectively. 23 Lastly, a recent systematic review confirms the initial good results of the TOT procedure, with an average probability of improvement in UI symptoms of 76.1%. 24 These studies are in line with our results, showing subjective improvement rates of SUI symptoms between 87 and 92%.

The literature also shows good results with the use of low-cost polypropylene mesh. Using the low-cost transobturator vaginal tape inside-out technique, a prospective evaluation of 59 women demonstrated 92% cure of SIU. 13 In another work also using the low cost vaginal mesh, now using the outside-in route, Elgamasy et al. 25 achieved 87.5% cure for SUI. Given the evidence of safety and efficacy of using low-cost vaginal tape, these have proven to be a good alternative, especially in the public health system with few financial resources, such as the hospital where our patients were operated.

The dispute about preoperative UDS is further justified due to it being an invasive, time-consuming examination that can cause pain in the patient. Not least, its potential to cause psychological discomfort, mainly through feelings of shame and anxiety, leads up to 20% of women to not want to undergo the exam again. 26 Finally, even if low, there is a possibility of lower urinary tract infection, with an incidence of ∼ 3%, even in healthy women. 27

The present study has many methodological issues that should be discussed. The group selection bias commonly affects retrospective studies and ours was no different. It is reasonable to question that patients who did not undergo UDS preoperatively had mild UI and symptoms of pure SUI. Thus, as it was not a randomized study, to mitigate the selection bias, we used propensity score matching to compare the groups, despite the overall characteristics of the patients being similar between the groups.

It was not possible to apply objective questionnaires for urinary continence assessment, and postoperative subjective parameters were used, which decreases the possibility of replicability of our results. In addition, the postoperative follow-up of our patients was not long, perhaps because, as we are a tertiary hospital, many patients with good postoperative evolution are referred early to follow-up at less complex health units.

## Conclusion

The results of the present retrospective study suggest that preoperative UDS had no impact on UI cure outcomes as well as on urinary storage symptoms after the transobturator tape procedure in women with UI and stress symptoms predominately. These data reinforce the hypothesis that perhaps UDS are unnecessary in women with uncomplicated SUI.
